# Supplementary material for: Identification of photoperiod-induced specific miRNAs in the adrenal glands of Sunite sheep (Ovis aries)
Source: Front Vet Sci. 2022 Jul 22;9:888207. doi: 10.3389/fvets.2022.888207 (PMC9354845; doi:10.3389/fvets.2022.888207)
Supplement: Supplementary file 1 [file Data_Sheet_1.ZIP › Supplementary file/miRDeep2 file.zip!/miRDeep2 file/Know_Structure/SP42A1/oar-mir-411b.pdf]

miRBase precursor : oar-mir-411b
Total read count : 1462
oar-miR-411b-5p read count : 1435
oar-miR-411b-3p read count : 27
remaining reads : 0

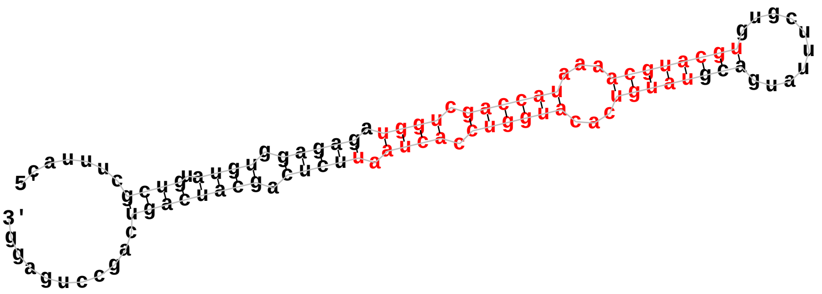

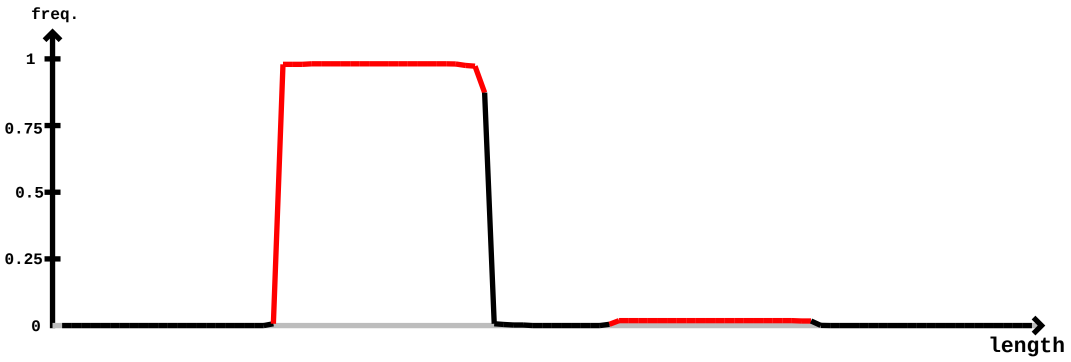


```
                                                         oar-miR-411b-3p
                        oar-miR-411b-5p
5'- cauuucgcuguauguggagagauggucgaccauaaaacguacgugugcuuuaugacguaugucacaugguccacuaauucucagcaucagucagccugagg   -3'    exp
    ......((((.((((.(((((.((((.((((((...((((((((..........))))))))...)))))).)))).))))).))))))))..........    reads     mm     sample
    .....................auggucgaccauaaaacguacg..........................................................    10        0      seq
    ......................uggucgaccauaaaacgu.............................................................    1         0      seq
    ......................uggucgaccauaaaacgua............................................................    8         0      seq
    ......................uggucgaccauaaaacguac...........................................................    4         0      seq
    ......................uggucgaccauaaaacguacg..........................................................    135       0      seq
    ......................uggucgaccauaaaacguacgu.........................................................    1267      0      seq
    ......................uggucgaccauaaaacguacgug........................................................    4         0      seq
    ......................uggucgaccauaaaacguacgugu.......................................................    3         0      seq
    .........................ucgaccauaaaacguacgugugc.....................................................    3         0      seq
    ........................................................guaugucacaugguccacua.........................    1         0      seq
    ........................................................guaugucacaugguccacuaau.......................    6         0      seq
    .........................................................uaugucacaugguccacua.........................    1         0      seq
    .........................................................uaugucacaugguccacuaau.......................    18        0      seq
    .........................................................uaugucacaugguccacuaauu......................    1         0      seq
```
